# Medical treatment of acquired uterine arteriovenous fistulae related to pregnancy: 2 case reports and literature review

**DOI:** 10.3389/fmed.2025.1584099

**Published:** 2025-08-05

**Authors:** Lingna Huang, Lini Qiu, Jinwen Zhang, Hui Ye, Xiumei Xiong

**Affiliations:** ^1^Department of Gynecology and Onbstetrics, Fujian Maternity and Child Health Hospital, College of Clinical Medicine for Obstetrics & Gynecology and Pediatrics, Fujian Medical University, Fuzhou, Fujian, China; ^2^School of Basic Medicine Sciences, Fujian Medical University, Fuzhou, Fujian, China

**Keywords:** uterine arteriovenous fistula, pregnancy-related, medical treatment, PSV, progesterone, GnRH-a

## Abstract

Uterine arteriovenous fistula (UAVF) is a rare form of uterine arteriovenous malformation, the most characteristic clinical symptom of which is intermittent vaginal bleeding. Currently, reports on the treatment of UAVF mainly focus on uterine artery embolization (UAE), high-intensity focused ultrasound (HIFU), and surgical treatment, with relatively few reports on medical conservative therapy. This article reports the process and outcomes of medical treatment for two cases of pregnancy-related acquired UAVF and summarizes and analyzes the indications and drug selection for UAVF medical conservative treatment based on the relevant literature.

## Introduction

Uterine arteriovenous fistula (UAVF) is a rare form of uterine arteriovenous malformation, with an unclear incidence rate in clinical practice. UAVF can be classified into congenital and acquired types based on etiology. Congenital UAVF is caused by the abnormal development of embryonic primitive blood vessels during the embryonic period and may be associated with other systemic vascular anomalies (1, 2). Acquired UAVF refers to vascular malformations caused by acquired factors such as pregnancy-related diseases, uterine procedures, infections, uterine tumors, and other factors (3). Owing to the rarity of this disease, diagnosing it can be quite challenging, especially if acquired UAVF is not identified in a timely manner, which may lead to significant vaginal bleeding and even life-threatening hemorrhagic shock. In early reports, hysterectomy and internal iliac artery or uterine artery ligation were the preferred treatment methods for UAVF (4, 5). With the advancements in technology, current reports in the literature have focused mainly on treatment methods such as uterine artery embolization (UAE), high-intensity focused ultrasound (HIFU), and surgical treatment (6–8), whereas relatively few reports on medical conservative treatment exist. This article reviews the treatment process of two cases of pregnancy-related acquired UAVFs and summarizes the efficacy and advantages of medical conservative treatment for UAVFs based on the domestic and foreign literature.

## Case 1

**Figure 1:**
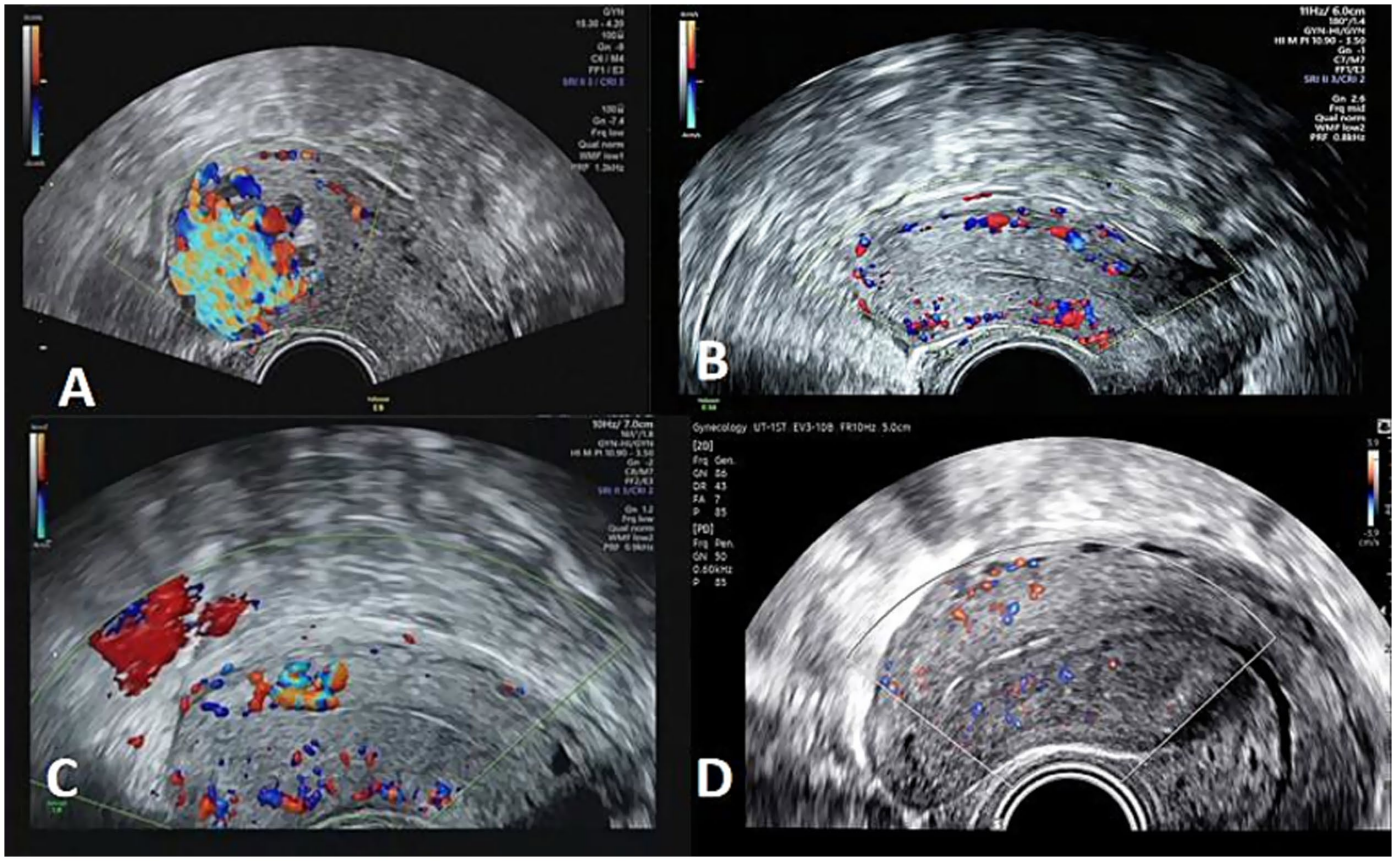
Ultrasound images of two patients before and after drug treatment. **(A,C)** Color Doppler ultrasound images of blood flow before treatment for patients in Patients 1 and 2, respectively. **(B,D)** Blood flow ultrasound images after treatment.

**Figure 2:**
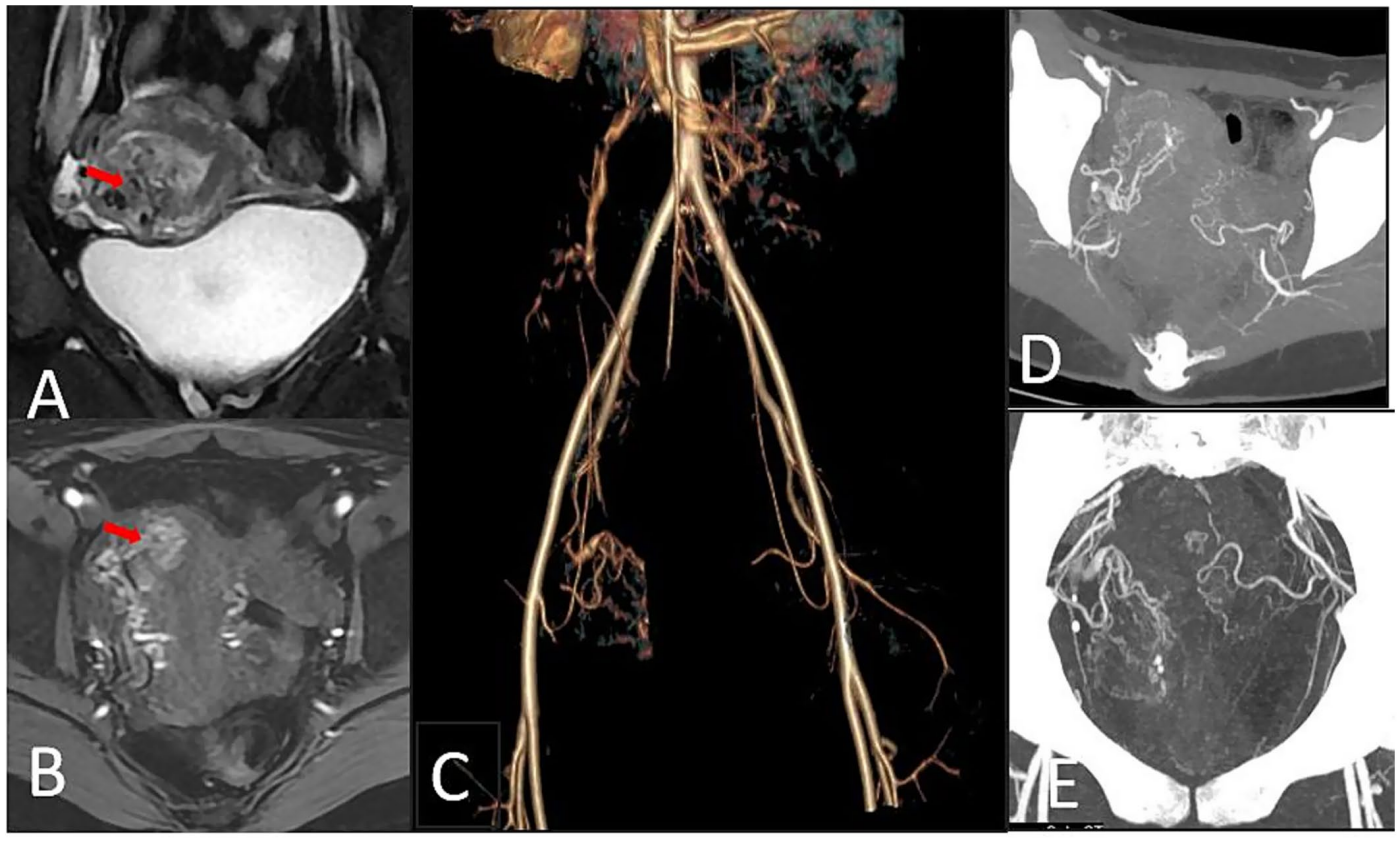
MRI and CTA images of two patients before drug treatment. **(A,B)** Coronal and dynamic scan images of Patient 1, which show early arterial development and a significant increase in the number of uterine veins on the right wall. **(C–E)** Postprocessed CTA images of Patient 2, which indicate that the right uterine vein appears earlier and significantly increases in size compared with the left.

A 31-year-old woman, G_3_P_1_, was admitted to the hospital on 12 April 2023 due to “uterine cavity lesions discovered 10 days after medication-induced abortion 25 days ago.” The patient experienced menarche at the age of 12. In 2019, she gave birth to a baby girl at full term through a normal delivery. In 2021, during the fifth month of pregnancy, she underwent induced labor and subsequent curettage due to “fetal brain protrusion.” In March 2023, at 13 weeks of pregnancy, she had to undergo an induced labor procedure due to “embryo cessation.” The patient underwent medical abortion with mifepristone and misoprostol 25 days before admission without undergoing a uterine curettage procedure. Following the abortion, she experienced recurrent vaginal bleeding, varying from light to heavy, with the heavier episodes being approximately twice her usual menstrual flow. Two weeks after induced abortion (2023.4.3), a follow-up ultrasound revealed an uneven echo in the fundus of the uterine cavity to the right anterior wall (measuring approximately 2.7 cm × 2.6 cm × 3.8 cm, with abundant blood flow signals inside). Serum beta-human chorionic gonadotropin (*β*-hCG) levels decreased as follows: 950.34 mIU/mL (3.31) → 650.874 mIU/mL (4.3) → 308.85 mIU/mL (4.6) → 102.79 mIU/mL (4.12). Further three-dimensional ultrasound (Figure 1A) revealed erosion of the anterior wall muscle layer in the uterine cavity (measuring approximately 3.4 cm × 2.0 cm × 3.6 cm, with unclear boundaries and abundant blood flow signals resembling a fireball, a left uterine artery peak systolic velocity (PSV) of 71.9 cm/s, and a right uterine artery PSV of 95.4 cm/s), suggesting a possible arteriovenous fistula or gestational trophoblastic tumor. Moreover, pelvic magnetic resonance imaging revealed a mixed signal mass in the uterine cavity with a small amount of bleeding (involving the local and deep muscle layers). Considering the medical history, a uterine arteriovenous fistula was suspected (Figures 2A,B). The serum *β*-hcg concentration was increased to 87.30 mIU/mL. The patient’s serum β-hCG level steadily decreased after induced abortion. When the results of color Doppler ultrasound and pelvic MRI are combined, gestational trophoblastic tumors are ruled out, leading to the diagnosis of uterine arteriovenous fistula. Following a comprehensive discussion on the pros and cons of different treatment methods, as well as the potential risks involved, the patient chose medication-based conservative treatment. On 19 April 2023, she received an injection of a gonadotropin-releasing hormone analog (GnRH-a) (3.75 mg intramuscular injection of leuprorelin acetate, once every 28 days) for three consecutive courses. She was also prescribed oral medroxyprogesterone acetate ethinyl estradiol tablets (II) concurrently. Regular monitoring revealed a continuous decrease in blood *β*-hCG levels: 34.32 mIU/mL (4.27) → 26.93 mIU/mL (5.5) → 10.66 mIU/mL (5.19) → <1.2 mIU/mL (6.2). After two cycles of medroxyprogesterone acetate ethinyl estradiol tablets (II), a follow-up three-dimensional color Doppler ultrasound on 21st June revealed uneven endometrial echo and significantly reduced blood flow in the anterior wall muscle layer (Figure 1B).

## Case 2

A 26-year-old woman, G_3_P_1_, was admitted with a chief complaint of “repeated vaginal bleeding for over a month after induced abortion, with heavy vaginal bleeding on 3 occasions.” She underwent an induced abortion at 24 weeks of pregnancy in 2019 due to fetal tetralogy of Fallot malformation. In 2021, she delivered a baby girl at full term via vaginal delivery. On 8 July 2023, at 15 weeks and 6 days of pregnancy, the embryo stopped developing, and the patient underwent a “Lifano abortion procedure.” Following the procedure, the uterus was not completely cleaned, resulting in intermittent light vaginal bleeding. On 29 July 2023, the patient experienced a significant amount of vaginal bleeding, bright red in color, approximately 2–3 times heavier than the usual amount of menstrual flow, which lasted for 1 day before stopping. Despite this, the patient did not seek further medical attention, as she thought it was just her menstrual period. On 3 August 2023, there was another episode of heavy vaginal bleeding, bright red in color, approximately 4–5 times heavier than the usual menstrual flow, accompanied by large blood clots. An emergency gynecological ultrasound revealed an irregular slightly hyperechoic area measuring approximately 5.0 cm × 2.2 cm × 3.2 cm in the uterine cavity, with color Doppler flow signals indicating blood flow from the posterior wall. On 4 August 2023, hysteroscopic resection of the intrauterine contents was performed under general anesthesia. The postoperative pathological result report showed that the substances within the uterine cavity were degenerated decidual-like tissues interspersed with individual highly degenerated villi. After discharge, there was intermittent light vaginal bleeding. A follow-up gynecological ultrasound on 14 August 2023 revealed a small amount of fluid in the uterine cavity. On 16 August 2023, without any apparent cause, there was a significant amount of fresh red vaginal bleeding, approximately 2–3 times heavier than the usual menstrual flow. Treatment with oxytocin nasal spray reduced vaginal bleeding. On 17 August 2023, there was another episode of significant vaginal bleeding, with the color being bright red and the amount being approximately 4–5 times heavier than the usual menstrual flow. A follow-up gynecological ultrasound (Figure 1C) revealed an uneven hypoechoic area with an abundant blood flow signal in the posterior wall of the uterus (PSV 82.2 cm/s). Considering the medical history, a “possible uterine arteriovenous fistula” was suspected. A routine blood examination revealed the following: hemoglobin 87 g/L, *β*-HCG 5.63 U/mL, and fibrinogen 1.75 g/L. Pelvic computed tomography angiography (CTA) (Figures 2C–E) revealed irregularly distributed disordered vascular shadows in the posterior upper wall of the uterus (some extending into the uterine cavity), suggesting vascular lesions and possibly an AVF. The patient was treated with mifepristone acetate combined with levonorgestrel ethinyl estradiol tablets and reexamined with three-dimensional color Doppler ultrasound (Figure 1D) after one cycle: slight uterine cavity effusion, a serum *β*-hCG concentration <1.2 mIU/mL, and oral levonorgestrel ethinyl estradiol tablets for two cycles.

After 12 months of treatment, we followed up with two patients. Patient 1 was in good general condition posttreatment, with no major bleeding during the treatment period. Currently, her menstrual cycle is regular, and her periods and flow are normal. Patient 2 is currently in good general condition, with no major bleeding during the treatment. Her current menstrual cycle is regular, but the amount of menstrual flow has significantly decreased. She is planning to undergo further hysteroscopy examination before attempting to conceive (Table 1).

**Table 1:** Summary of the diagnosis and treatment of the two patients.

Case#	Age	Maternity history	History of gynecological surgery (including this one)	Symptom	HCG value (mIU/mL) upon discovering UAVF	Psv (cm/s)	Treatment plan	Follow-up results
1	31	G3P1	Drug-induced abortion once, followed by uterine curettage once	Recurrent vaginal bleeding, varying from light to heavy	102.79	Left: 71.9 Right: 95.4	GnRH - *α* x3 + drospirenone ethinylestradiol tablets for two cycles	Menstrual cycle regularity, normal menstrual period, and menstrual flow
2	26	G3P1	Induced abortion twice during mid-term pregnancy, both followed by curettage surgery	Minor vaginal bleeding, accompanied by frequent vaginal bleeding resembling opening and closing	5.63	82.2	GnRH - *α* x1 + drospirenone ethinylestradiol tablets for three cycles	Normal menstrual cycle, reduced menstrual flow, currently in a state of preparing for pregnancy

## Discussion

Both patients in this study were diagnosed with pregnancy-related acquired UAVF through a combination of color Doppler ultrasound and imaging studies. The clinical presentation of UAVF is mainly irregular vaginal bleeding, with the most common characteristic being intermittent vaginal bleeding. In severe cases, patients may experience life-threatening massive bleeding or intra-abdominal bleeding that endangers their lives (9). Acquired UAVF is usually associated with pregnancy, particularly as a result of cesarean section or uterine curettage procedures that expose blood vessels and infiltration of blood vessel walls by malignant uterine tumors (10). Easton-Carr et al. suggested that mechanical dilation or curettage may easily stimulate reactive blood vessel formation and that a high level of human chorionic gonadotropin or high estrogen status can stimulate angiogenesis and vascularization, which may all be mechanisms triggering UAVF (11, 12). Kulshrestha et al., on the other hand, proposed that an increased immune response, which occurs postsurgery, plays a crucial role in abnormal angiogenesis (13)(DSA) is the gold standard for diagnosing UAVF, it is not commonly used in routine clinical practice because of its invasive nature. As a result, ultrasound examination is the preferred imaging modality (14). Uterine arteriovenous fistulas are visible on color Doppler ultrasound, with blood flow appearing in varying directions and colors mixing together. Measuring the PSV and resistance index (RI) can effectively reflect the hemodynamic characteristics of uterine arteriovenous fistulas (15). However, ultrasound examination may lack specificity, and in such cases, a combination of CTA and MRI may be required for a definitive diagnosis.

Many studies have reported that UAE, HIFU, and surgical treatments have achieved good results in the treatment of UAVFs. In particular, UAE is considered a first-line treatment for UAVF patients with recurrent bleeding, severe bleeding, or unstable hemodynamics who wish to preserve fertility because of its minimally invasive nature (16–18). However, some studies have noted that UAE can also cause ovarian dysfunction, thinning of the endometrium, and increase the risk of miscarriage, premature birth, fetal growth restriction, or postpartum hemorrhage (19, 20). Additionally, UAE, HIFU, and various surgical treatments are associated with a certain degree of trauma and high treatment costs. Therefore, for patients with minimal vaginal bleeding, relatively small lesions, and stable hemodynamics, medical therapy is economically effective and non-invasive, indicating that it has broad prospects for application (10).

## Indications for conservative medical treatment

International evidence-based medicine does not yet have clear guidelines for the conservative medical treatment of UAVF. Currently, medical therapy is recommended for patients with UAVF who are not pregnant or have relatively small lesions after pregnancy termination and minimal vaginal bleeding. Additionally, the blood flow characteristics of color Doppler ultrasound can provide some guidance for clinical treatment. Timmerman et al. (21) proposed classifying women diagnosed with uterine arteriovenous malformation into three groups: (1) those with life-threatening uterine bleeding, confirmed by color Doppler ultrasound and angiography; (2) those with heavy vaginal bleeding and arteriovenous malformation (AVM) features on Doppler ultrasound but not confirmed by angiography, and (3) those with mild symptoms and vascular abnormalities on ultrasound. Women at risk of life-threatening bleeding (groups 1 and 2) require immediate hysterectomy for hemostasis. For patients who still desire fertility, UAE or hysteroscopic electrocoagulation can be considered. Women with mild or asymptomatic symptoms (group 3) can be managed conservatively with medication or expectant treatment. Timmerman also suggested that the risk of bleeding can be determined by the PSV value on color Doppler ultrasound. The PSV is more valuable than the PI and RI in assessing the risk of bleeding: if the PSV is ≥ 0.83 m/s, it indicates a high potential risk of bleeding; if the PSV is < 0.83 m/s, it suggests a low risk of bleeding from vascular malformations; and if the PSV is < 0.39 m/s, it is considered relatively safe. Therefore, Taneja et al. suggested that, if the lesion of a UAVF detected by pelvic ultrasound is relatively small, with a PSV of 40–60 cm/s and minimal vaginal bleeding, medical treatment can be attempted. The two patients in this study met the above criteria, so medical therapy was chosen, and the treatment process progressed smoothly with a good outcome (10). Compared with UAE and other surgical treatments, UAVF medical therapy is easier to obtain, with advantages such as being non-invasive, cost-effective, and having minimal impact on ovarian function without affecting future fertility. According to Rosen et al. (22), a meta-analysis of 32 studies involving a total of 121 cases of AVM treated with medication concluded that the overall success rate for medical treatment was 88%. Medical management of symptomatic uterine AVM is a reasonable approach in well-selected patients who are hemodynamically stable and have reliable follow-up.

## Types of drugs used for conservative treatment

Commonly used drugs in clinical practice for treating UAVF include potent progestins, gonadotropin-releasing hormone agonists (GnRH-a), combination oral contraceptives, and methotrexate, which can be used alone or in combination.

### Progesterone

Progesterone can cause atrophy and thinning of endometrial glands by binding to receptors, thereby reducing bleeding. When given for prolonged durations, progesterone helps to maintain avascular status. On the other hand, following a miscarriage, there is a high estrogen state, whereas progesterone is a potent antiestrogen. Progesterone can stimulate the activity of 17-*β*-hydroxysteroid dehydrogenase and sulfotransferase (these two enzymes work together to convert estradiol to estrone sulfate, which is rapidly cleared from the body), thereby inhibiting the induction of estrogen on its own receptors and further antagonizing the effects of estrogen. The effectiveness of highly effective progesterone treatment for uterine arteriovenous malformations has been validated in multiple clinical trials (23). Taneja et al. proposed through a prospective trial that oral medroxyprogesterone acetate is a safe and effective novel oral medication that can replace embolization or surgical treatment for bleeding caused by uterine arteriovenous malformations following a miscarriage (10).

### Estrogen

The treatment of UAVF with estrogen is approached from two different angles. According to Siple et al. (24), estrogen can stimulate endometrial growth, repair bleeding blood vessels, and effectively stop bleeding. Conversely, Nonaka et al. argued that high estrogen levels can lead to vascular endothelial proliferation and endometrial changes, which are the underlying causes of UAVF (25). Therefore, when estrogen is used to treat UAVF, it is recommended that it be administered at low doses.

### Gonadotropin-releasing hormone agonist

Previous studies have shown that vascular malformations outside the pelvic cavity can significantly improve or disappear after bilateral oophorectomy, suggesting a possible association between vascular malformations and sex hormones (26). Many scholars have reported successful cases of treating UAVF and achieving successful pregnancies with GnRH-a (27). GnRH-a primarily reduces estrogen secretion, resulting in endometrial thinning, decreased endometrial blood supply, increased uterine artery resistance, and the promotion of vascular malformation sclerosis for therapeutic purposes (28).

### Compound oral contraceptives

Combined oral contraceptives include a combination of low-dose estrogen and progestin, which are commonly used to treat UAVFs with clear efficacy. The high-efficiency progestin in combination with oral contraceptive pills shrinks the endometrium, reduces bleeding, and inhibits the synthesis of estrogen receptors, increasing resistance to the body’s “high estrogen state.” Moreover, the low-dose synthetic estrogen in combination with oral contraceptive pills promotes endometrial proliferation, repairs bleeding blood vessels, synergizes with progestin, and stabilizes the endometrium. Estrogen and progestin also work together to have a “multitarget” effect, not only by acting on the endometrium but also by suppressing the hypothalamic–pituitary–ovarian axis, thereby inhibiting ovarian function (27, 29).

### Danazol

Danazol can reduce uterine blood flow and is used to treat UAVF, but its exact mechanism is still unclear. Danazol may directly act on the uterine artery or have secondary effects on the endometrium, affecting smaller resistance vessels (i.e., subendometrial vessels) and thereby reducing uterine blood flow to achieve therapeutic goals. Cases of successful treatment of uterine arteriovenous malformations with danazol were reported by Tak et al. and Takeuchi et al. (30, 31).

In addition, misoprostol, methotrexate, aromatase inhibitors, oxytocin, and tranexamic acid have also been used in combination for the treatment of UAVF (3, 32, 33). Regardless of the medication selected for treatment, close monitoring in outpatient settings is necessary. If there is persistent bleeding or an increase in vaginal bleeding during treatment, uterine artery embolization (UAE) or surgical intervention may still be needed.

## Summary

The treatment of UAVF is comprehensively considered based on the patient’s age, severity of bleeding, fertility needs, and lesion size. For non-pregnant and post-pregnant patients with relatively small UAVF lesions, stable hemodynamics (PSV < 83 cm/s), and less vaginal bleeding, drug therapy can be considered.

## Data Availability

The original contributions presented in the study are included in the article/supplementary material, further inquiries can be directed to the corresponding author.
